# A case of an infant suspected as IMAGE syndrome who were finally diagnosed with MIRAGE syndrome by targeted Mendelian exome sequencing

**DOI:** 10.1186/s12881-018-0546-4

**Published:** 2018-03-05

**Authors:** Yoon-Myung Kim, Go Hun Seo, Gu-Hwan Kim, Jung Min Ko, Jin-Ho Choi, Han-Wook Yoo

**Affiliations:** 1 Department of Pediatrics, Asan Medical Center Children’s Hospital, University of Ulsan College of Medicine, 88, Olympic-ro 43-gil, Songpa-Gu, Seoul, 05505 South Korea; 2 Medical Genetics Center, Asan Medical Center Children’s Hospital, University of Ulsan College of Medicine, Seoul, South Korea; 3 Department of Pediatrics, Seoul National University Children’s Hospital, Seoul, South Korea

**Keywords:** Congenital adrenal hypoplasia, *SAMD9*, MIRAGE syndrome

## Abstract

**Background:**

Adrenal hypoplasia is a rare congenital disorder, which can be classified into a non-syndromic form, without extra-adrenal features, and a syndromic form, with such features. Despite biochemical and molecular genetic evaluation, etiologic diagnosis cannot be performed in many patients with adrenal hypoplasia.

**Case presentation:**

The patient in this case was a boy born at 31 weeks of gestation with a weight of 882 g (< 3rd percentile) to non-consanguineous parents. Genital examination showed micropenis and bilateral cryptorchidism. On the third day of life, he manifested hypotension with high urine output, hyponatremia, hyperkalemia, hypernatriuria, high plasma adrenocorticotropic hormone level, and high plasma renin activity, suggesting acute adrenal insufficiency. The serum 17α-hydroxyprogesterone level was normal. Adrenal insufficiency improved following administration of hydrocortisone and 9α-fludrocortisone, but the patient died of recurrent infection at 4 months of age. He was suspected as IMAGE (Intrauterine growth restriction, Metaphyseal dysplasia, Adrenal hypoplasia congenita, and Genital anomalies) syndrome. However, no mutation in *CDKN1C* was identified. Targeted exome sequencing using the TruSight One Sequencing Panel (Illumina) identified a heterozygous mutation of c.2944C > T (p.R982C) in exon 3 in *SAMD9*.

**Conclusion:**

This report describes the first Korean case of MIRAGE syndrome. The patient presented with severe primary adrenal insufficiency, intrauterine growth retardation, and recurrent infection. *SAMD9* mutation should be considered in patients who present with adrenal hypoplasia and extra-adrenal phenotypes.

## Background

Major etiologies of adrenal insufficiency in children include impaired steroidogenesis, adrenal hypoplasia, familial glucocorticoid deficiency (FGD), FGD-like disorders, and adrenal destruction [1, 2]. Congenital adrenal hyperplasia accounts for 80% of the causes of pediatric adrenal insufficiency [1]. Recent advances in molecular genetic technologies have enabled identification of a variety of rare causative genes in patients with primary adrenal insufficiency [2, 3]. However, 20%–60% of patients with primary adrenal insufficiency remain genetically undiagnosed [2, 4]. Causative genes for primary adrenal hypoplasia include *NR0B1* and *NR5A1,* which play a key role in human adrenal development [5, 6], and *CDKN1C,* which is known to cause IMAGE (Intrauterine growth restriction, Metaphyseal dysplasia, Adrenal hypoplasia congenita, and Genital anomalies) syndrome [7, 8]. Mutations in *MC2R, MRAP, NNT, TXNRD2,* and *AAAS* are related to adrenal hypoplasia as part of an adrenocorticotropic hormone (ACTH) resistance syndrome [1]. Adrenal hypoplasia occurs secondarily to defects in transcription factors involved in pituitary development, such as *HESX1, LHX4,* and *SOX3* [9], and rare syndromes such as Meckel-Gruber syndrome, Pena-Shokeir syndrome, hydrolethalus syndrome, Galloway-Mowat syndrome, and Pallister-Hall syndrome [3]. In 2016, mutations in *SAMD9* were identified in patients with adrenal hypoplasia with extra-adrenal features such as intrauterine growth restriction, recurrent infections, gonadal and bone marrow failure, a condition designated as MIRAGE (Myelodysplasia, Infection, Restriction of growth, Adrenal hypoplasia, Genital phenotypes, and Enteropathy) syndrome [10]. To date, a total of 21 patients with the syndrome, with 16 different mutations, have been reported [10–12].

It is difficult to make a correct diagnosis in patients with syndromic adrenal hypoplasia owing to the diverse genetic etiologies and overlapping clinical and biochemical features. The present report describes a patient with MIRAGE syndrome who presented with intrauterine growth retardation, adrenal insufficiency, and recurrent infection and had been initially suspected as having IMAGE syndrome.

## Case presentation

The patient in this case was the first child born to non-consanguineous parents with no family history of adrenal insufficiency. He was born at 31 weeks of gestation by Cesarean section, owing to severe oligohydramnios and fetal heart rate variability. At birth, the patient had symmetric intrauterine growth retardation with a weight of 882 g (< 3rd percentile), a length of 35 cm (< 3rd percentile), and a head circumference of 24 cm (< 3rd percentile). On physical examination, profound skin hyperpigmentation, frontal bossing, low-set ears, bilateral cryptorchidism, and micropenis were noted.

The patient underwent mechanical ventilation immediately after birth owing to respiratory difficulty. Indomethacin was used on the sixth day of life to treat patent ductus arteriosus with pulmonary edema, and closure was eventually achieved. Three days after birth, the patient manifested hypotension, polyuria (8.1 mL/kg/h), hyponatremia (128 mmol/L), hyperkalemia (6.0 mmol/L), hypernatriuria (104 mEq/L), and an increased fractional excretion of sodium (6.2%). Plasma ACTH level was tremendously elevated to 4670 pg/mL (normal range, 10–60 pg/mL), with plasma cortisol at 5.6 μg/dL (normal range, 2.5–9.1 μg/dL), plasma renin activity at 217 ng/mL/h (normal range, < 2.8 ng/mL/h), and plasma aldosterone at 55.1 pg/mL (normal range 190–1410 pg/mL), suggesting severe adrenal crisis. However, plasma 17α-hydroxyprogesterone level was 6.0 ng/mL (normal range at gestational age of 31 weeks, 1–172 ng/mL). A stress dose of intravenous hydrocortisone (95 mg/M^2^/day), fludrocortisone acetate (0.02 mg/day), and sodium chloride (8 mEq/kg/day) were administered. Subsequently, the patient’s serum electrolytes, ACTH level, and blood pressure normalized, and the skin hyperpigmentation also showed improvement. The plasma ACTH level decreased to 82.7 pg/mL after 1 week of therapy and was maintained between 20 pg/mL and 40 pg/mL during the follow-up period. Abdominal ultrasonography revealed non-visualization of both adrenal glands and the right testis. The left testis was located in the patient’s abdominal cavity, and a right inguinal hernia was observed. Brain ultrasonography was normal.

Mild thrombocytopenia, ranging from 70,000 to 150,000/mm^3^, and normocytic anemia, ranging from 6.4 to 10.8 g/dL, manifested during the first week of life. Thrombocytopenia and anemia resolved spontaneously 1 month after birth, with the counts maintained over 300,000/mm^3^ and 10 g/dL, respectively. Blood cultures were negative for bacteria, and there was no evidence of congenital infections including toxoplasmosis, rubella, cytomegalovirus (CMV), or herpes simplex virus. Serum IgM level was below 4.5 mg/dL (normal range, 7.3 ± 3.3 mg/dL). The thymic shadow was visible on chest radiography. At 1 month of age, an elevation in C-reactive protein (CRP) up to 3 mg/dL and leukocytosis (white blood cell [WBC] count of 35,400/mm^3^) were observed. Intravenous antibiotics, including vancomycin and amikacin, were administered under the diagnosis of neonatal sepsis. *Serratia marcescens (S. marcescens)* (50,000/mL) and *Enterococcus faecium* (> 100,000/mL) were cultured in urine. However, no bacteria were isolated in blood cultures or cerebrospinal fluid. Follow-up urine culture was clear after administration of antibiotics for 4 days; however, CRP and WBC count were still elevated to 2–3 mg/dL and 15,000–20,000/mm^3^, respectively. Two weeks later, the patient manifested apnea and decreased oxygen saturation, with diffuse haziness in both lungs on chest radiography. Despite administration of antibiotics including vancomycin, imipenem/cilastatin, and amphotericin B, pneumonia, and respiratory difficulty gradually worsened, ultimately requiring the resumption of mechanical ventilation. Urine CMV culture, blood CMV polymerase chain reaction, and blood CMV IgG and IgM tests were all positive. Blood and urine CMV cultures were converted to negative after 3 weeks of intravenous ganciclovir. After 3 weeks of mechanical ventilation, *S. marcescens* was identified from cultured endotracheal aspirates (100,000,000/mL) and urine (80,000/mL). Despite antibiotic treatment, pneumonia was aggravated, and *S. marcescens* was also identified in blood cultures when the patient was 4 months old. CRP and WBC count rose to 25.9 mg/dL and 32,100/mm^3^, respectively. The pneumonia and sepsis exacerbated and the patient died of septic shock at 4 months of age.

**Fig. 1:**
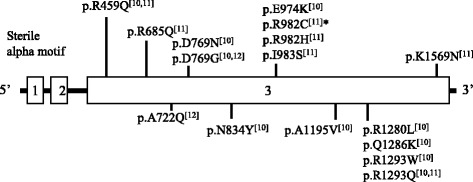
Mutation spectrum of *SAMD9* gene in patients with MIRAGE syndrome. *, a mutation in the current study

**Fig. 2:**
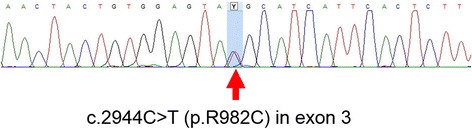
Partial sequence of the *SAMD9* gene. Sanger sequencing of *SAMD9* identified a heterozygous mutation of c.2944C > T (p.R982C) in exon 3

The patient’s karyotype performed at age one month was 46, XY without any chromosomal structural abnormality. Under suspicion of X-linked adrenal hypoplasia congenita or IMAGE syndrome, genetic analysis was performed. However, no mutation was identified in the *NR0B1* or *CDKN1C* genes by Sanger sequencing. Therefore, targeted exome sequencing was performed using genomic DNA extracted from peripheral blood leukocytes. Written informed consent was obtained for exome sequencing from all parents and the study was approved by the local ethics committee. The exome was captured using the Trusight One Panel (Illumina Inc., San Diego, CA, USA), which enriches a 12-Mb region spanning 4813 genes, and was sequenced on the NextSeq platform (Illumina Inc.). The mean depth of coverage was 114× and approximately 98% of targeted bases were read more than 10×. Reads were aligned to the hg19 human reference genome. The result was identification of a heterozygous missense variant of c.2944C > T (p.R982C) in exon 3 in the *SAMD9* gene, which was reported to be pathogenic with gain-of-function effect [11] (Fig. 1). This variant was not found in normative population databases including 1000Genomes browser (http://phase3browser.1000genomes.org/) and Exome Aggregation Consortium Browser (http://exac.broadinstitute.org/) and predicted to be damaging by in silico prediction programs, including PolyPhen-2 (http://genetics.bwh.harvard.edu/pph2/; score of 0.616) and Sorting Intolerant From Tolerant (http://sift.jcvi.org/; score of 0). This mutation was confirmed by Sanger sequencing using custom-designed primers (Fig. 2). Genetic analysis of parents could not be performed to confirm the de novo nature of the variant because the parents’ blood samples were not available.

## Discussion and conclusions

This report describes a case of MIRAGE syndrome with a heterozygous missense mutation in *SAMD9* in a patient who was initially suspected as having IMAGE syndrome. Recent advances in molecular genetic technologies have enabled the identification of new causative genes in patients with primary adrenal insufficiency of unknown etiology, but some patients remain genetically undiagnosed. To avoid missing the diagnosis of this newly recognized disease, it is necessary to carefully examine the extra-adrenal features.

Common features of MIRAGE syndrome include recurrent invasive infection, restriction of growth, adrenal hypoplasia, and genital phenotypes [10, 11]. Other clinical features are preterm delivery, respiratory distress syndrome, and frontal bossing [11]. Chronic diarrhea with colonic dilatation was observed in almost all patients in previous reports [10, 11]. Clinical characteristics of patients with MIRAGE syndrome in previous studies and the present report are summarized in Table 1. In the case described here, the patient manifested no gastrointestinal symptoms or additional features such as cerebellar hypoplasia, hydrocephalus, or short phalanges.

**Table 1:** Clinical characteristics of patients reported to have *SAMD9* mutation and adrenal insufficiency

	Narumi et al. (2016) [10]	Buonocore et al. (2017) [11]	Present study
Number of patients	11	8	1
Male:Female	7:4	8:0	Male
IUGR	11	8	+
Preterm delivery	NA	8 (32 weeks)	+ (31 weeks)
Adrenal insufficiency	11	7	+
Ambiguous genitalia	7	8	+
Monosomy 7 or 7q	2	5	–
Recurrent infection	10	7	+
Age at death (years)	1.5 ± 1.6 (range, 0.3–5)	0.6 ± 0.6 (range, 0–1.8)	0.3
Survival	3	2	–

*IUGR* intrauterine growth restriction, *NA* not assessed

Myelodysplastic syndrome with mosaic monosomy 7 or monosomy 7q by the loss of the other allele on chromosome 7 has been founded in as many as seven of 19 patients, suggesting a gain of survival advantage [10, 11]. Secondary somatic loss-of-function mutations of *SAMD9* have been identified in four of eight patients, and this type of mutation also seems to be part of a survival strategy [11]. More than half of the identified patients died before 2 years of age owing to invasive infection including sepsis, meningitis, and fungal infections [10, 11]. Postmortem analysis of two patients revealed thymic hypoplasia with a decrease in the number of cortical lymphocytes [10]. Two patients with mosaic monosomy 7 or monosomy 7q who underwent bone marrow transplantation were alive at the time of the report [11]. Discovering the pathogenesis of recurrent invasive infection in patients with MIRAGE syndrome might help increase survival, considering that almost all patients in the studies to date have died as a result of infection [10, 11].

*SAMD9* protein products are involved in the endosome system and might influence the growth factor signal transduction pathway [10, 13, 14], resulting in profound growth inhibition in in vitro analysis [10, 11]. *SAMD9* protein products contain a sterile alpha motif (SAM) domain near the N-terminal region and a widely expressed putative protein interaction module involved in interactions with proteins, DNA, and RNA. [13, 15]. Several phenotypes of *SAMD9* disorders seem to be causally related to the wide expression of the *SAMD9* gene, including its expression in adrenal glands, colon, bone marrow, liver, immune system, lung, and testis [11, 16]. Biallelic inactivating mutations in *SAMD9* cause normophosphatemic familial tumoral calcinosis, leading to the hereditary form of dystrophic calcification in the skin [13, 17, 18]. Downregulation of *SAMD9* has also been found in mouse models with aggressive fibromatosis, breast cancer, colon cancer, and non-small cell lung cancer [15, 19]. In contrast, overexpressing *SAMD9* mutations are presumed to be related to growth-restricting conditions due to the gain of function [10, 11].

The differential diagnosis of congenital adrenal hypoplasia includes X-linked adrenal hypoplasia congenita, *NR5A1* mutation, Xp21 contiguous gene deletion syndrome, IMAGE syndrome, and ACTH insensitivity syndrome [1, 3]. Even within the same disease, the degree of clinical manifestations varies widely. Extra-adrenal features must be evaluated carefully to perform a diagnosis in patients with syndromic adrenal hypoplasia. IMAGE syndrome and MIRAGE syndrome share common features such as intrauterine growth restriction, adrenal hypoplasia, and genital anomalies, which could lead to confusion in diagnosis. Other characteristic features of IMAGE syndrome such as metaphyseal dysplasia, relative macrocephaly, and hypercalciuria have not been reported in MIRAGE syndrome [7]. The presence of recurrent invasive infection or chronic diarrhea suggests MIRAGE syndrome [10, 11].

In conclusion, SAMD9 mutation should be considered in patients who present with adrenal hypoplasia and extra-adrenal phenotypes such as intrauterine growth restriction and recurrent infection. Further research is necessary to investigate this extremely rare cause of primary adrenal insufficiency with extra-adrenal features.

## Abbreviations

AAAS: ACHALASIA-ADDISONIANISM-ALACRIMA SYNDROME
ACTH: Adrenocorticotropic hormone
CDKN1C: CYCLIN-DEPENDENT KINASE INHIBITOR 1C
CMV: Cytomegalovirus
CRP: C-reactive protein
FGD: Familial glucocorticoid deficiency
HESX1: HOMEOBOX GENE EXPRESSED IN ES CELLS
Ig: Immunoglobulin
IMAGE: Intrauterine growth restriction, Metaphyseal dysplasia, Adrenal hypoplasia congenita, and Genital anomalies
LHX4: LIM HOMEOBOX GENE 4
MC2R: MELANOCORTIN 2 RECEPTOR
MIRAGE: Myelodysplasia, Infection, Restriction of growth, Adrenal hypoplasia, Genital phenotypes, and Enteropathy
MRAP: MELANOCORTIN 2 RECEPTOR ACCESSORY PROTEIN
NNT: NICOTINAMIDE NUCLEOTIDE TRANSHYDROGENASE
NR0B1: NUCLEAR RECEPTOR SUBFAMILY 0, GROUP B, MEMBER 1
NR5A1: NUCLEAR RECEPTOR SUBFAMILY 5, GROUP A, MEMBER 1
SAMD9: STERILE ALPHA MOTIF DOMAIN-CONTAINING PROTEIN 9
SOX3: SRY-Box 3
TXNRD2: THIOREDOXIN REDUCTASE 2
WBC: White blood cell
